# An improved integrative GFP-based vector for genetic engineering of *Parageobacillus thermoglucosidasius* facilitates the identification of a key sporulation regulator

**DOI:** 10.1186/s13568-023-01544-9

**Published:** 2023-05-08

**Authors:** Marie Millgaard, Gonzalo Nahuel Bidart, Ivan Pogrebnyakov, Alex Toftgaard Nielsen, Ditte Hededam Welner

**Affiliations:** The Novo Nordisk Foundation Center for Biosustainability, Building 220, Kemitorvet, 2800 Kgs. Lyngby, Denmark

**Keywords:** Genetic engineering, Thermophile, *Parageobacillus thermoglucosidasius*, Shuttle vector, Sporulation

## Abstract

*Parageobacillus thermoglucosidasius* is a thermophilic Gram-positive bacterium, which is a promising host organism for sustainable bio-based production processes. However, to take full advantage of the potential of *P. thermoglucosidasius*, more efficient tools for genetic engineering are required. The present study describes an improved shuttle vector, which speeds up recombination-based genomic modification by incorporating a thermostable sfGFP variant into the vector backbone. This additional selection marker allows for easier identification of recombinants, thereby removing the need for several culturing steps. The novel GFP-based shuttle is therefore capable of facilitating faster metabolic engineering of *P. thermoglucosidasius* through genomic deletion, integration, or exchange. To demonstrate the efficiency of the new system, the GFP-based vector was utilised for deletion of the *spo0A* gene in *P. thermoglucosidasius* DSM2542. This gene is known to be a key regulator of sporulation in *Bacillus subtilis*, and it was therefore hypothesised that the deletion of *spo0A* in *P. thermoglucosiadius* would produce an analogous sporulation-inhibited phenotype. Subsequent analyses of cell morphology and culture heat resistance suggests that the *P. thermoglucosidasius* ∆*spo0A* strain is sporulation-deficient. This strain may be an excellent starting point for future cell factory engineering of *P. thermoglucosidasius*, as the formation of endospores is normally not a desired trait in large-scale production.

## Introduction

Most industrial production is still relying heavily on fossil fuels to meet the ever-increasing demands for commodities such as fuels, chemicals, and plastics. However, growing concerns regarding the environmental impact of fossil fuel consumption has caused a shift in interest towards the development of more sustainable production processes (Ögmundarson et al. 2020). Microbial fermentations present a promising solution, as these kinds of strategies allow for the conversion of renewable feedstocks into valuable resources. Despite this, large-scale microbial production processes are still relatively uncommon on the global market, as they struggle to compete with fossil-fuel based alternatives. One way to counter this issue would be through the development of more efficient production hosts. As such, increased focus has been given to the development of optimised cell factories that reliably and efficiently convert substrate into product (Hermann et al. 2007; Biddy et al. 2016).

Thermophilic bacteria have long served as vital sources of thermostable proteins and catalysts (Haki and Rakshit 2003). However, thermophiles also hold a lot of potential to be favourable hosts for other kinds of large-scale productions, as there are multiple benefits to running microbial productions at higher temperatures (Zeikus 1979; Haki and Rakshit 2003; Bhalla et al. 2013; Najar and Thakur 2020). Notably, cooling costs of large-scale fermentations are lowered, and the risk of contamination from mesophiles is reduced (Zeldes et al. 2015; Krüger et al. 2018). Moreover, volatile products can be readily recovered from high-temperature fermentation broths, meaning that volatile compound production would avoid the challenge of product inhibition and toxicity, while having significantly eased downstream processing (Zeikus 1979; Najar and Thakur 2020).

Among thermophiles, the two Gram-positive genera of *Geobacillus* and *Parageobacillus* are particularly notable, as they have already provided promising candidates for the production of various enzymes, chemicals, and biofuels (Taylor et al. 2009; Najar and Thakur 2020). A prominent candidate is *P. thermoglucosidasius*, an obligately thermophilic endospore-forming bacteria with an optimal growth temperature of 61–63 °C (Najar and Thakur 2020). There currently exist a number of tools for genetic engineering of *Geobacillus* and *Parageobacillus* species, including different transformation strategies (Kananavičiūtė and Čitavičius 2015), CRISPR/Cas9-based genome editing (Lau et al. 2021), gene expression tools (Pogrebnyakov et al. 2017), and a variety of plasmid-based shuttle vectors (Taylor et al. 2008; Cripps et al. 2009; Kananavičiūtė and Čitavičius 2015; Madika et al. 2022). As one of the thermophiles receptive to genetic modification, *P. thermoglucosidasius* has already successfully been engineered for production purposes, particularly for ethanol (Cripps et al. 2009; Zhou et al. 2016; Sheng et al. 2017). However, compared to its more well-studied mesophilic counterparts, the *P. thermoglucosidasius* genetic engineering toolbox is still considerably underequipped. In order to take full advantage of the potential of *P. thermoglucosidasius* as a production organism, it is critical to develop more efficient strategies for genetic manipulation. Here, we demonstrate an improved shuttle-vector design for performing genomic modifications in *P. thermoglucosidasius*.

### Shuttle vectors for genetic engineering in *P. thermoglucosidasius*

Traditionally, the generation of specific deletion and insertion mutants in *P. thermoglucosidasius* relies on homologous recombination to perform allelic exchange using integrative shuttle vectors (Taylor et al. 2008; Cripps et al. 2009). The two-step allelic exchange through homologous recombination requires cloning the flanking regions of the target into an appropriate integrative shuttle vector, such as the pUCG18 developed for *P. thermoglucosidasius* by Taylor et al. (Taylor et al. 2008).

Generally, the shuttle vector is transformed into the desired *P. thermoglucosidasius* strain, most commonly through either electroporation or conjugation (Kananavičiūtė and Čitavičius 2015). Following this, the native homologous recombination machinery of the cell may integrate the vector into the *P. thermoglucosidasius* chromosome at the target site matching the deletion allele (flanking regions). As the shuttle vector is unable to replicate at high temperatures due to its temperature-sensitive origin of replication, the resulting recombinants can be selected by growing the strain at high temperature in the presence of an antibiotic matching the selective marker encoded by the vector. Once the successful recombinants have been obtained, yet another recombination event can be initiated by subculturing without antibiotics. As there is no longer any selection pressure present, the cells are no longer required to retain the resistance cassette in their chromosome. The lack of selective pressure thereby allows the natural homologous recombination system of the cells to recombine the integrated homologous flanks with their matching sequences once again. During this second recombination step, the vector backbone is lost and, depending on which flanks recombine, it can result in either a revertant strain identical to the original, or the targeted mutant strain. Given that the only selectable marker present in the currently available vectors for *P. thermoglucosidasius* is antibiotic resistance, second recombinants must be identified through their loss of resistance. This is usually achieved through a series of plating steps, in which the cells are grown with and without antibiotics present in order to determine their sensitivity. Finally, once the second recombinants have been identified, successful mutants can be discerned from wildtype revertants through means such as colony PCR and sequencing.

One major issue with the traditional shuttle vector strategy is the high number of time-consuming steps required to perform genetic modifications. However, as seen with recombination-based vectors developed for species such as *Pseudomonas putida* (Calero et al. 2016; Volke et al. 2021), the addition of fluorescent markers to the vector backbone can allow for more time-efficient detection of recombinants. As such, this study presents an improved genome-editing vector for *P. thermoglucosidasius* which—in addition to an antibiotic resistance marker—carries a thermostable variant of sfGFP under a strong constitutive promotor (P7). The presence of sfGFP within the vector simplifies the generation of *P. thermoglucosidasius* mutant strains by allowing for direct identification of second recombination events through absence of fluorescence—thereby removing the need to perform multiple culturing steps in order to screen for loss of antibiotic resistance. To demonstrate the efficiency of the improved vector, a scarless deletion of the *spo0A* gene in *P. thermoglucosidasius* DSM2542 was carried out in this study.

### Sporulation in *P. thermoglucosidasius*

Sporulation is a strategy that is undertaken by some bacteria, including *P. thermoglucosidasius*, as a natural response to unfavourable conditions—most commonly in the form of nutrient starvation. Compared to vegetative cells, the resulting dormant endospores are usually remarkably resistant to extreme stresses, such as high temperatures, ultraviolet radiation, and a variety of chemical solvents (Nicholson et al. 2000; Errington 2003). In large-scale productions, sporulation often presents a recurring problem, as it prompts the cells to funnel resources towards the development of endospores, and consequently drains away nutrients, limits the yield, and slows the fermentation process. In addition, the endurance of endospores may allow them to survive common sterilisation techniques, thereby greatly increasing the risk of contaminations within the factory setting. For this reason, contract fermentation organisations typically do not accept sporulating organisms.

**Fig. 1:**
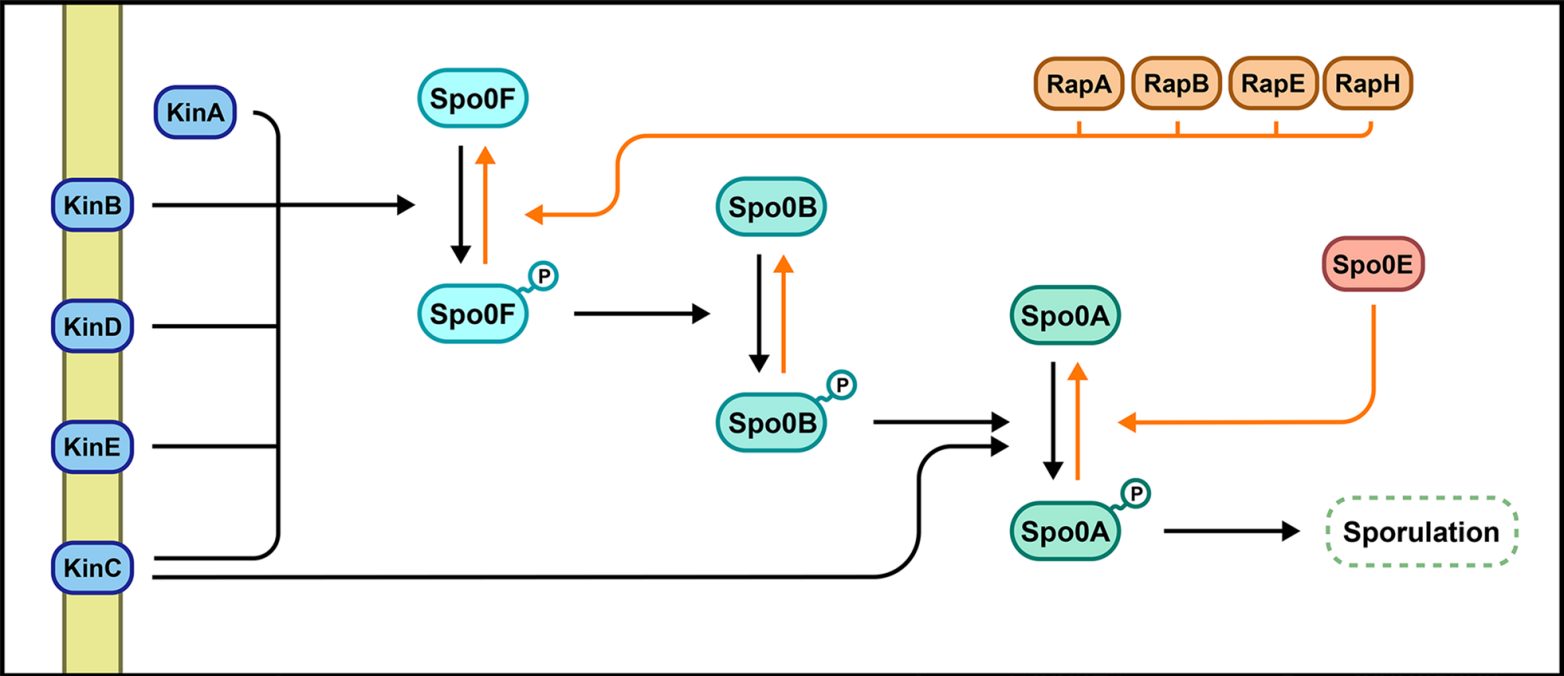
A simplified overview of the sporulation initiation process in *B. subtilis*. Sporulation is initiated once high levels of phosphorylated Spo0A protein is present in the cell. Sporulation upregulating effects are marked in black, while downregulating effects are marked in orange. Spo0A phosphorylation is primarily facilitated by phosphotransferases Spo0F and Spo0B. Major upregulations of the phosphorelay are performed by histidine kinases KinA-E in response to environmental stresses. As counters, phosphatases RapA, B, E, and H as well as Spo0E are capable of dephosphorylating specific steps of the pathway

The development of a sporulation-deficient *P. thermoglucosidasius* would significantly improve its applicability as a production organism. To demonstrate the efficiency of the presented deletion system, it was therefore decided to carry out the deletion of the *spo0A* gene in *P. thermoglucosidasius* DSM2542. This specific gene was chosen due to its critical role in the initiation of the sporulation pathway, as observed in the more well-studied *Bacillus subtilis* (Errington 2003; Piggot and Hilbert 2004). In *B. subtilis*, the entry to the sporulation pathway is primarily controlled by the activation of the transcriptional key regulator Spo0A through phosphorylation. Once activated, Spo0A is known to directly regulate the expression of approximately 121 genes, including the activation of genes necessary for initiating the sporulation pathway (Molle et al. 2003). While low levels of activated Spo0A typically leads to behaviours such as biofilm formation and cannibalism, high levels of activated Spo0A is required before the cell is able to enter the sporulation developmental program (Piggot and Hilbert 2004; Tan and Ramamurthi 2014). The activation of Spo0A is facilitated by phosphotransferases Spo0F and Spo0B, which in turn are governed by five autophosphorylating histidine kinases (KinA-KinE) that respond to environmental stimuli and upregulate the phosphorelay system accordingly. Another prominent regulation method is performed by phosphatases, which are known to downregulate the pathway through the dephosphorylating of Spo0F (RapA, B, E, and H) or by dephosphorylating Spo0A directly (Spo0E) (Fig. 1). These are only a few examples of the numerous regulators that *B. subtilis* employ to manage the levels of phosphorylated Spo0A, as sporulation initiation must be carefully regulated to ensure that the cell only commits to the laborious process of sporulation when circumstances require it (Jiang et al. 2000; Errington 2003; Piggot and Hilbert 2004; Higgins and Dworkin 2012; Tan and Ramamurthi 2014).

In the case of *P. thermoglucosidasius,* the sporulation pathway is currently not as well-characterised as that of *B. subtilis*. However, homologues of Spo0A, Spo0B, and Spo0F have been identified in the genome of *P. thermoglucosidasius*, suggesting that the sporulation initiation of this species is operated through a similar regulatory system. Notably, the *spo0A* gene of *P. thermoglucosidasius* shares approximately 80% homology with its counterpart in *B. subtilis*, indicating that they maintain similar functions. Under the assumption that the two species carry analogous sporulation systems, the removal of *spo0A* should significantly affect the ability of *P. thermoglucosidasius* to sporulate (Wang et al. 2020). As such, the present study aims to document the impact the deletion of *spo0A* will have on spore-development in *P. thermoglucosidasius*.

## Materials and methods

### Strains, plasmids, and media

The strains and plasmids used in this study are listed in Table 1. The *P. thermoglucosidasius* DSM2542 strains were routinely grown at 60 °C under agitation in SPY medium. SPY contains, per litre: 16 g soy peptone, 10 g yeast extract, and 5 g NaCl, with pH adjusted to 6.8. In addition, *P. thermoglucosidasius* strains were plated on Trypticase Soy Agar (TSA) plates (Becton Dickinson, US) unless stated differently.

**Table 1:** Strains and plasmids used in the present study

Strain	Relevant genotype or properties	Source
*P. thermoglucosidasius* DSM2542/BGSC ID 95A1	Wildtype isolate	Bacillus Genetic Stock Center (USA)
Pth::pMM7	DSM2542::pMM7 Kan^R^	This work
Pth ∆*spo0A*	DSM2542 Δ*spo0A*	This work
*E. coli* DH5α™	F- Φ80*lac*ZΔM15 Δ(*lac*ZYA-*arg*F) U169 *rec*A1 *end*A1 *hsd*R17(rk-, mk +) *pho*A *sup*E44 *thi*-1 *gyr*A96 *rel*A1 λ-	ThermoFisher Scientific
*E. coli* DH5α-λ*pir*	F- φ80*lac*Z∆M15 ∆(*lac*ZYA-*arg*F)U169 *rec*A1 *end*A1 *hsd*R17(rk-, mk +) *pho*A *sup*E44 *thi*-1 *gyr*A96 *rel*A1 λ-*pir* lysogen of DH5α	Lab collection

Plasmid	Description	Source
pMTL61110	*E. coli*/*P. thermoglucosidasius* integrative shuttle vector*,* Thermosensitive replicon	Sheng et al. 2017
pIP7	Vector carrying sfGFP	﻿Pogrebnyakov et al. 2017
pGB-sfGFP	pMTL61110 carrying P7-sfGFP	This work
pGB-sfGFP-best	pGB-sfGFP (F11V, T13P, N39D, A179A)	This work
pMM7	pGB-sfGFP-best carrying flanking regions of *spo0A*	This work

*E. coli* DH5α was used as host in cloning experiments and grown in lysogeny broth (LB) medium at 37 °C under agitation. *E. coli* DH5α and *P. thermoglucosidasius* DSM2542 transformants were all selected with kanamycin (6.25 mg/L and 12.5 mg/L respectively).

Sporulation was induced by culturing *P. thermoglucosidasius* past the exponential phase in Thermophile Minimal Medium (TMM) supplemented with 3 g/L yeast extract. TMM is adapted from Fong et al. (Fong et al. 2006) and contained following sterile solutions, per litre: 930 mL Six Salts Solution (SSS), 40 mL of 1 M MOPS solution (pH = 8.2), 10 mL of 1 mM FeSO_4_ in 0.4 M tricine, 10 mL of 0.132 M K_2_HPO_4_, 10 mL of 0.953 M NH_4_Cl, 0.5 mL of 1 M CaCl_2_, 0.5 mL of trace elements solution, and 1 mL of Wolfe’s vitamin solution, with the final pH adjusted to 6.8. SSS contains, per litre: 4.95 g NaCl, 1.45 g Na_2_SO_4_, 0.25 g KCl, 0.04 g KBr, 1.85 g MgCl_2_·6H_2_O, and 0.89 g NaNO_3_. The trace elements solution contains, per litre: 1 g FeCl_3_·6H_2_O, 0.18 g ZnSO_4_·7H_2_O, 0.12 g CuCl_2_·2H_2_O, 0.12 g MnSO_4_·H_2_O, and 0.18 g CoCl_2_·6H_2_O. Finally, Wolfe’s vitamin solution contains, per litre: 10 mg Pyridoxine HCl, 5 mg Thiamine HCl, 5 mg Riboflavin, 5 mg Nicotinic acid, 5 mg Ca-D-( +)phantothenate, 5 mg p-Aminobenzoic acid, 5 mg Thiotic acid (Dithiolane Pentanoic acid), 2 mg Biotin, 2 mg Folic acid, and 0.1 mg Vitamin B12.

### *P. thermoglucosidasius* competent cells

*P. thermoglucosidasius* DSM2542 cells were made competent before the transformation of the plasmid. This was accomplished by inoculating the strain in 50 mL of SPY medium. The culture was incubated at 60 °C while shaking until it reached an OD_600_ of approximately 1.5. Subsequently, the culture was diluted to an OD_600_ of 0.5 in a new flask containing 30 mL of fresh SPY. This flask was then incubated at 60 °C while shaking, until it reached an OD_600_ of approximately 1.7. The culture was then placed on ice for 10 min, after which it was split into two aliquots, which were spun down at 4,000 g in a 4 °C centrifuge. The aliquots were each spun down and resuspended four times in 15, 10, 10, and 5 mL of ice-cold electroporation buffer (0.5 M mannitol, 0.5 M sorbitol, 10% glycerol). Following this, both aliquots were resuspended in 2 mL of electroporation buffer. The final suspensions were then transferred as 60 µl aliquots to tubes pre-chilled on dry ice. The competent cell aliquots were frozen at − 80 °C.

### Improvement of integrative shuttle vector pMTL61110

The shuttle-vector pGB-sfGFP-best was constructed using the vector pMTL61110 as a backbone template. P7-sfGFP was obtained from pIP7, and the fragments were assembled through USER cloning (New England Biolabs, US). USER cloning was performed by mixing all fragments and then supplying them with 1.2 µl of 10 × CutSmart Buffer (New England Biolabs, US), DNase/RNase-free water, and 1 µl of USER enzyme (New England Biolabs, US) to a total of 12 µl. The reaction was subsequently incubated at 37 °C for 25 min and then 25 °C for 25 min. Following this, 8 µl of DNase/RNase-free water was added to the reaction for a total volume of 20 µl, and 5 µl of the mixture was then directly transformed into chemically competent *E. coli* DH5α™ (Table 1).

To provide appropriate temperature stability and bright fluorescence in *P. thermoglucosidasius*, mutations in sfGFP (F11V, T13P, N39D, A179A) (Frenzel et al. 2018) were also introduced by PCR using USER cloning (New England Biolabs, US), with all primers listed in Table 2. All constructs were verified through a DNA sequencing service (Eurofins, Germany) before being transformed into electrocompetent *P. thermoglucosidasius* DSM2542 (see previous section).

**Table 2:** Primers used for construction and verification of pGB-sfGFP-best, pMM7, and the *P. thermoglucosidasius* ∆*spo0A* strain

No	Sequence 5ʹ-3ʹ	Description
Geo Vector backbone-For	AGTTTGUTGAAGATTAGATGCTATAATTGTT	Forward primer to amplify pGB backbone from pMTL61110
Geo Vector backbone-Rev	AGCCGGTTGUTTTGCCGGCCGGCCCTACTCTTTA	Reverse primer to amplify pGB backbone from pMTL61110
P7_sfGFP_Ter-For	ACAACCGGCUCCTTTTGCTCTTTCTTCT	Forward primer to amplify P7-sfGFP from pIP7
P7_sfGFP_Ter-Rev	ACAAACUGAGAAAAAGAAACAGAGGC	Reverse primer to amplify P7-sfGFP from pIP7
SeqIns_For	TTGCAATGTGGAATTGGG	Forward primer to amplify and sequence the sfGFP insert region in the vector
SeqIns_Rev	ATAACTCGTCTTCCTAAGCATCC	Reverse primer to amplify and sequence the sfGFP insert region in the vector
F11V/T13P-For	ATTCTGGUGGAACTGGATGGTGATGTC	Forward primer for introduction of F11V/T13P in sfGFP
F11V/T13P-Rev	ACCAGAAUAG**G**GACGA**C**ACCAGTGAACAGCTCTTCGCC	Reverse primer for introduction of F11V/T13P in sfGFP
N39D-For	ACT**G**ATGGUAAACTGACGCTGAAGTTCATCTG	Forward primer for introduction of N39D in sfGFP
N39D-Rev	ACCAT**C**AGUTGCGTCACCTTCACCCTC	Reverse primer for introduction of N39D in sfGFP
A179A-For	AGCTGGC**C**GAUCACTACCAGCAAAACACT	Forward primer for introduction of A179A in sfGFP
A179A-Rev	ATC**G**GCCAGCUGCACGCTGCCATCCTCCA	Reverse primer for introduction of A179A in sfGFP
23	ACCCGGGGUTCCTCTAG	Forward primer to amplify pMM7 backbone fragment from pGB-sfGFP-best
24d	AATTCGUAATCATGGTCATATGGATACAGCG	Reverse primer to amplify pMM7 backbone fragment from pGB-sfGFP-best
11M2	GGCCGCTGTATCCATATGACCATG	Forward primer for sequencing of homologous arms region in pMM7
12	GTTGTAAAACGACGGCCAGTGC	Reverse primer for sequencing of homologous arms region in pMM7
PNJ1241	ACGAATUTAATATCACTCGATGCTTAAAATCAATCTCATCAACC	Forward primer to amplify *spo0A* left homologous arm from gDNA
PNJ1242	AGCCGCUAATATCAACGGTTTAAATGTCATG	Reverse primer to amplify *spo0A* left homologous arm from gDNA
PNJ1243	AGCGGCUCTATGCAAAAAAAGCTAAATTTATGACGTTTTTCACG	Forward primer to amplify *spo0A* right homologous arm from gDNA
PNJ1244	ATCCCCGGGUCCGCATTGGATTATATATCCGAGATTCTGC	Reverse primer to amplify *spo0A* right homologous arm from gDNA
PNJ1245	CAGTTCCGTATCTGTATGTCTGTCCTTAAATCG	Forward primer for sequencing of *spo0A* genomic region
PNJ1246	GGAAAAAAATCGAAAACATGGATGATCTTTCC	Reverse primer for sequencing of *spo0A* genomic region

Underlined sequences indicate overhangs designed for assembly with other fragments during USER cloning, while changed nucleotides are marked with bold

### Construction of *spo0A* deletion vector (pMM7)

A shuttle vector pMM7 was constructed for the targeted deletion of *spo0A* in *P. thermoglucosidasius* DSM2542. The fragments were assembled through USER cloning (New England Biolabs, US), as described in previous section. A full list of primers used for the amplification of fragments is shown in Table 2. Backbone fragments were amplified using the temperature-sensitive vector pGB-sfGFP-best as template, while the homologous arms (of 750 bp each) were amplified from *P. thermoglucosidasius* DSM2542 gDNA. All fragments were purified using a NucleoSpin Gel and PCR kit (Macherey–Nagel, Germany). The plasmid was assembled through USER cloning, after which they were directly transformed into chemically competent *E. coli* DH5α-λ*pir* (Table 1). After recovery, the transformed cells were plated onto LB agar plates containing 6.25 mg/L kanamycin. Following incubation at 37 °C, colony PCR was performed with primer set 11M2/12 (Table 2) in order to identify colonies containing correctly assembled plasmid. The PCRs were performed using *OneTaq* Quick-Load 2X Master Mix with Standard Buffer (New England Biolabs, US), in accordance with manufacturers’ protocol. Positive colonies were purified using a NucleoSpin Plasmid kit (Macherey–Nagel, Germany), and the correct assembly of the purified plasmid was confirmed through sequencing (Eurofins, Germany) with primers 11M2 and 12.

### Deletion of *spo0A*

60 µl of competent *P. thermoglucosidasius* cells were mixed with 2.5 µl of pMM7. The cell-plasmid mixture was then transferred into an ice-cold electroporation cuvette. The cuvette was placed in an electroporation device and shocked with an exponential pulse and the parameters set to a voltage of 2500 V, capacitance of 10 µF, and resistance of 600 Ω. The mixture was then immediately transferred to 1 mL of SPY supplemented with 1% glycerol and pre-heated to 52 °C. The cells were allowed to recover for 3 h at 52 °C while shaking, after which they were spun down at 3,000 g and plated onto TSA plates supplied with 12.5 mg/L kanamycin. The plates were then incubated overnight at 52 °C.

The integration of the plasmid was initiated by inoculating successfully transformed colonies from the TSA-kanamycin plates into 2 mL SPY with 12.5 mg/L kanamycin. The cultures were left to incubate overnight at 62 °C while shaking. Next day, the cultures were streaked onto TSA plates containing 12.5 mg/L kanamycin, and the plates were allowed to incubate overnight at 60 °C. Following this, the colonies were screened for successful integrations through colony PCR with the primer sets PNJ1245/12 and PNJ1246/11M2 (Table 2). To perform colony PCR on *P. thermoglucosidadius*, colonies were first suspended in 20 µl of 20 mM NaOH and heated to 95 °C for 10 min. After being cooled to room temperature, the suspensions were then amplified using *OneTaq* Quick-Load 2X Master Mix with Standard Buffer (New England Biolabs, US), in accordance with manufacturers’ protocol.

Colonies that had successfully integrated pMM7 were selected for deletion of *spo0A*. The colonies were inoculated in 2 mL of SPY and left to incubate overnight at 60 °C while shaking. The following two days, all cultures were regularly transferred to fresh medium. This was done by passaging 500 µl of each culture into 1.5 mL of fresh pre-heated SPY during the morning and afternoon. On the afternoon of the second day, the cultures were diluted and seeded onto TSA plates which were then left to incubate overnight at 60 °C. The resulting colonies were observed under blue light. Non-fluorescent colonies were selected for colony PCR with the primer set PNJ1245/PNJ1246. Colonies displaying a short band with the predicted length were selected, and the knockout was later confirmed via sequencing.

### Growth assay

In order to observe any significant changes to the growth patterns and sporulation induction of *P. thermoglucosidasius* DSM2542 following the knockout of *spo0A*, a growth assay was performed. Here, the ∆*spo0A* strain and wildtype strain were each inoculated into 4 mL of SPY and left to incubate overnight at 60 °C while shaking. Next morning, the pre-cultures were each inoculated to an OD_600_ of 0.05 in three separate shake flasks containing 30 mL of TMM medium supplied with 3 g/L yeast extract.

After inoculation, the cultures were allowed to incubate for 24 h at 60 °C while shaking. Samples were collected at 0.5, 1, 2, 3, 4, 5, 6, 7, 8, and 24 h. The OD_600_ of each sample was measured, and microscopy was performed on samples collected at the 2-, 6-, 8-, and 24-h time points.

### Microscopy

Before microscopy, 1% agarose was used to prepare agar pads on glass slides. The microscopy slides were then each prepared by transferring 5 µl of pure culture onto an agar pad and covering it with a glass coverslip. Phase contrast microscopy was performed with a Leica DM4000 B microscope (Leica Microsystems, Germany) rigged with a 63 × oil immersion objective. Images were captured with a Leica DFC300 FX camera (Leica Microsystems, Germany) utilising the Leica Application Suite software, version 4.12.0 (Leica Microsystems, Germany).

### Heat resistance assay

The purpose of this assay was to examine the heat resistance of *P. thermoglucosidasius* wildtype and ∆*spo0A* cultures that have been subjected to nutrient starvation. Spores are expected to be resistant, while sporulation deficient strains should not survive heat treatment. The ∆*spo0A* strain and wildtype strain were each inoculated three times in 3 mL of TMM supplied with 3 g/L yeast extract. They were left to incubate for 24 h at 60 °C while shaking. Following this, the OD_600_ of the cultures were measured, and samples were collected for microscopy (see previous section). 500 µl of each culture was transferred to new tubes, which were then heated to 100 °C for 40 min while shaking at 1200 rpm. Next, 100 µl of each boiled culture was inoculated into tubes containing 2900 µl SPY. Additionally, 100 µl of each unboiled culture was inoculated in the same manner. The tubes were incubated at 60 °C while shaking. OD_600_ measurements were made after 24, 48, and 72 h of incubation.

## Results

### Construction of improved shuttle vector

To facilitate faster genetic engineering in *P. thermoglucosidasius*, we decided to develop an integrative shuttle vector that incorporates green fluorescence as a secondary selective marker, alongside kanamycin resistance. To this end, a particular thermostable variant of sfGFP (Frenzel et al. 2018) was selected, which has been evolved in *P. thermoglucosidasius* DSM2542 and displays an 885-fold increase in fluorescence at 60 °C in this strain, compared to that of the original sfGFP published by Pedelacq et al. (Pédelacq et al. 2006; Frenzel et al. 2018).

**Fig. 2:**
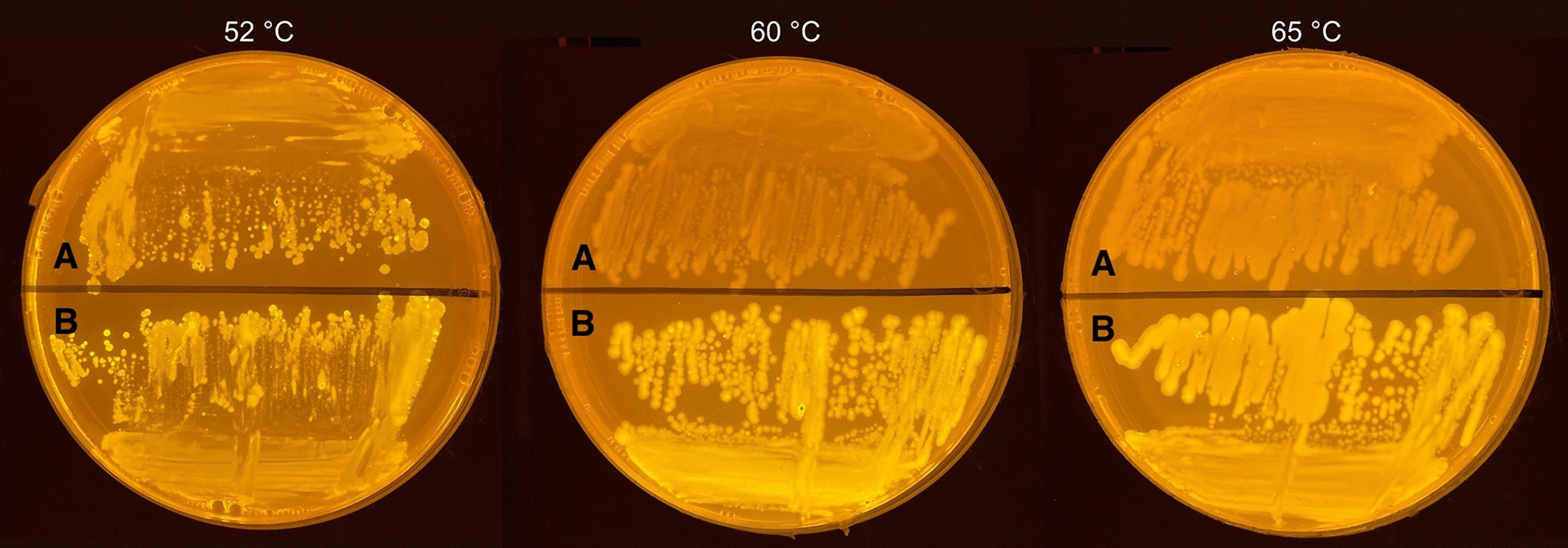
*P. thermoglucosidasius* DSM2542 cells transformed with pGB-sfGFP (**A**) and pGB-sfGFP-best (**B**) as viewed under blue light. Cells expressing the sfGFP variant evolved in *P. thermoglucosidasius* DSM2542 retain their fluorescence at high temperatures

The vector pIP7 (Pogrebnyakov et al. 2017), carrying a non-thermostable variant of sfGFP, was selected as template for the precursor P7-sfGFP. pIP7 was first subcloned into the current integrative shuttle vector pMTL61110 (Sheng et al. 2017), producing the pGB-sfGFP plasmid. Following this, site-directed mutagenesis was applied to introduce the four mutations required for the desired thermostable sfGFP variant (F11V, T13P, N39D, and A179A GCT → GCC), resulting in the final pGB-sfGFP-best shuttle vector. When transformed with pGB-sfGFP-best, *P. thermoglucosidasius* DSM2542 remained fluorescent when cultured up to 65 °C (Fig. 2), confirming previous findings (Frenzel et al. 2018).

### Generation and verification of *spo0A* knockout

In microbial production, sporulation is generally considered an undesirable trait, as the formation of endospores tends to deplete resources from production pathways and increase the risk of contamination. To improve *P. thermoglucosidasius* as a production host, this study therefore aimed to develop a sporulation-free strain by deleting the *spo0A* gene, which is known to be a key regulator of sporulation in *B. subtilis*.

**Fig. 3:**
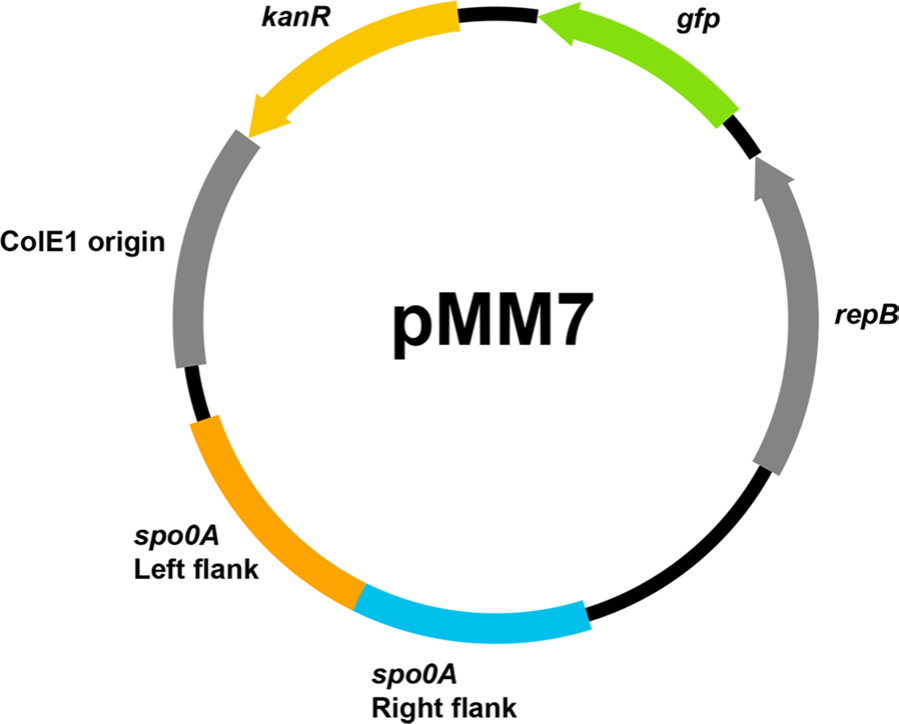
The basic structure of the pMM7 *spo0A* deletion plasmid. It contains Gram-positive replication protein *repB*, and Gram-negative ColE1 origin, a kanamycin resistance cassette, the temperature-optimised sfGFP gene, and a region containing the homologous flanks that match the upstream and downstream regions of the *spo0A* target site

**Fig. 4:**
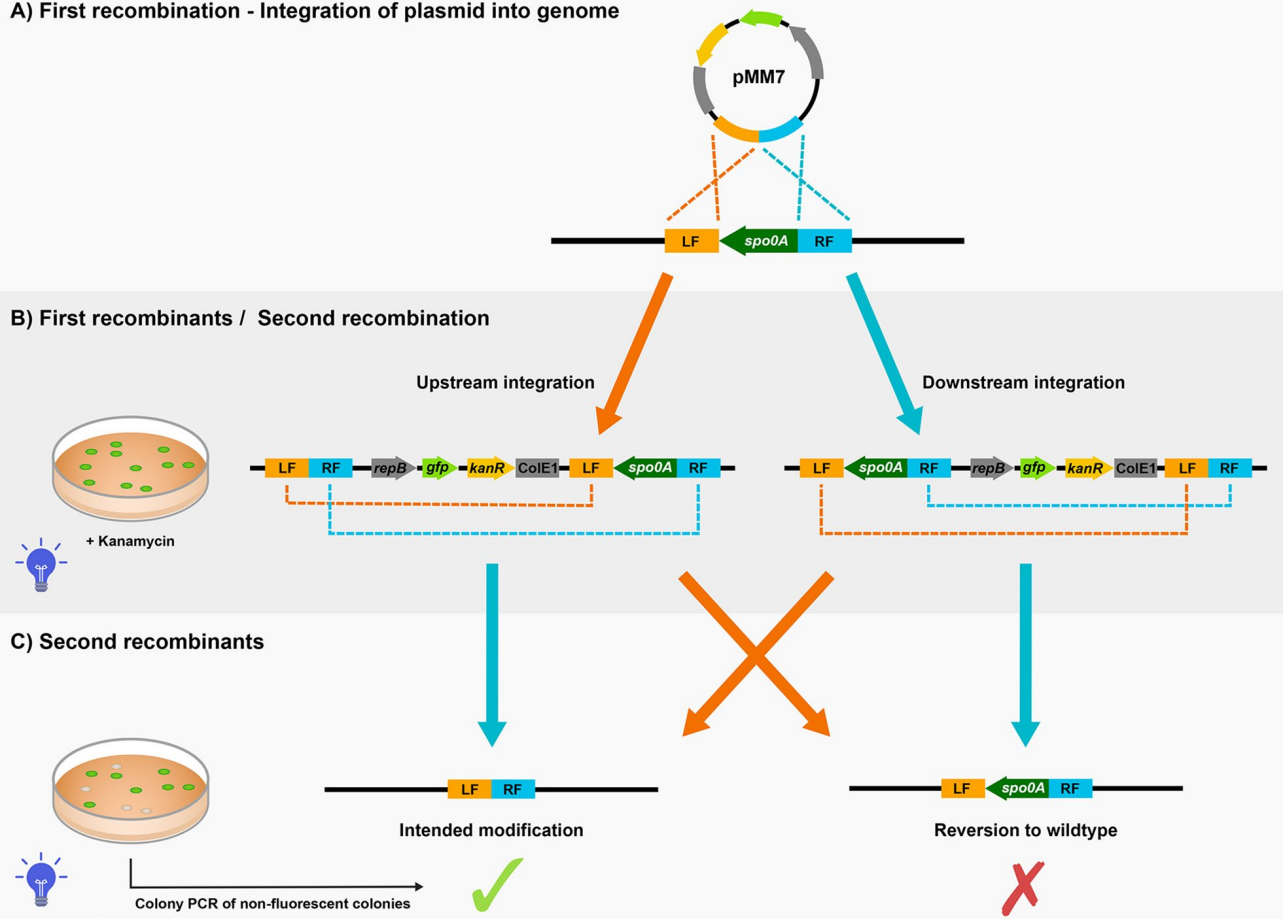
An example of the mechanism behind the generation of genetic modifications in *P. thermoglucosidasius* using the pMM7 *spo0A* deletion vector, as constructed from the improved shuttle vector design presented in this study. **A** Initially, pMM7 is integrated into the genome through homologous recombination at the target site. The successful recombinants are selected by culturing the cells at high temperatures with kanamycin. As the Gram-positive replication of pMM7 is temperature-sensitive, the cells will be forced to integrate the plasmids in order to retain kanamycin resistance. **B** Due to the presence of sfGFP, successful recombinants will fluoresce green when exposed to blue light. These first recombinants are subcultured without kanamycin for several days, during which some cells will eventually recombine the homologous regions in the vector-originated DNA and the genome for the second time. This results in the loss of the vector DNA. **C** After the second recombination, the sfGFP has been lost alongside the plasmid DNA, and successful second recombinants can therefore be identified by their lack of fluorescence. The second recombination can produce two outcomes, dependent on which two homologous flanks combined. They can either revert to wildtype or incorporate the intended modification into the strain. In the case of this study, the intended modification was the deletion of sporulation regulator *spo0A*

The deletion of *spo0A* was facilitated by the pMM7 vector, which was constructed from the improved shuttle vector pGB-sfGFP-best and carries the flanking regions of the *spo0A* target gene (Fig. 3). The method for performing the deletion of *spo0A* can be found in the “Materials and Methods” section, and the full mechanism behind the deletion can be viewed in Fig. 4.

**Fig. 5:**
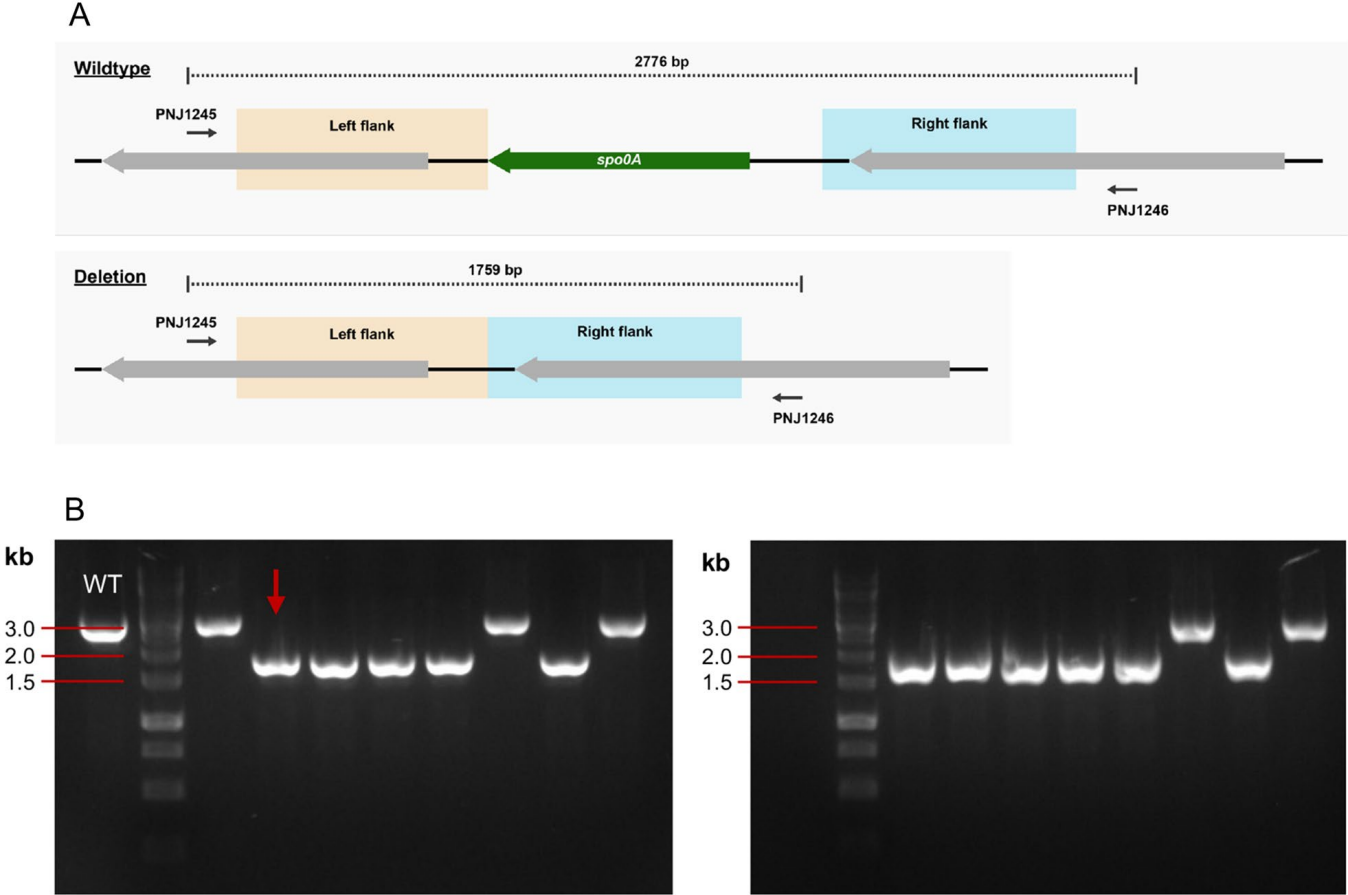
**A** The genomic composition of the *spo0A* region in *P. thermoglucosidasius* in the wildtype strain and following the deletion of *spo0A*. **B** Colony PCR of the *spo0A* region (primer set PNJ1245/PNJ1246) performed on second recombinants, as identified through lack of fluorescence. Note how some colonies have only looped out the pMM7, thus reverting to wildtype (matching wildtype control ‘WT’). The remaining colonies have looped out *spo0A* alongside pMM7, and the resulting bands are therefore significantly shorter. The band of the ∆*spo0A* colony selected for further verification and testing is marked with an arrow

Following the completion of the ∆*spo0A* gene deletion process, a few percent of the plated colonies were non-fluorescent. Of these, 16 colonies were selected for verification through colony PCR. Utilising the primer set PNJ1245/PNJ1246, wildtype revertant colonies should produce a band of approximately 2800 bp, same as the wildtype. In comparison, colonies that successfully deleted the gene would produce a band of approximately 1800 bp (Fig. 5A). Of the 16 selected colonies, 5 were determined to be wildtype revertants, while the remaining 11 colonies displayed the predicted shorter band (Fig. 5B). From these 11 colonies, one was selected for sequencing of the *spo0A* genomic region. The sequencing results confirmed the knockout, and this strain was then used in the subsequent growth and heat resistance assays.

### Growth characterization of *P. thermoglucosidasius* ∆*spo0A*

**Fig. 6:**
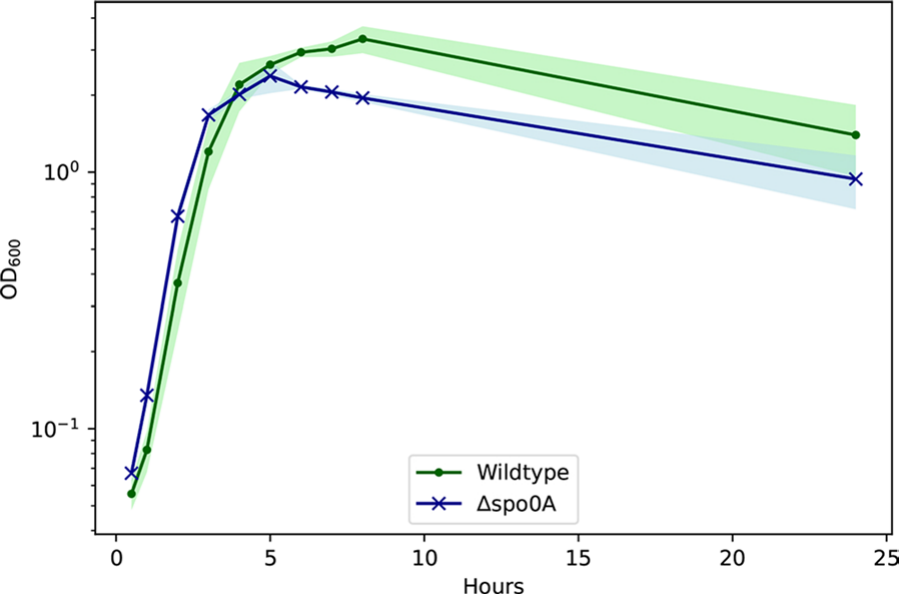
Growth curves over 24 h for the *P. thermoglucosidasius* ∆*spo0A* and wildtype strain. It can be seen how their growth is quite comparable, although the ∆*spo0A* strain appear to decrease in OD_600_ faster than the wildtype

Shortly after the inoculation of the pre-culture into fresh TMM medium supplemented with yeast extract, both the *P. thermoglucosidasius* ∆*spo0A* and wildtype cultures exhibited rapid growth. This growth continued for several hours, with very similar growth patterns between the two strains (Fig. 6).

Around the 5-h mark, growth was generally beginning to stagnate for both strains. However, while the OD_600_ of the ∆*spo0A* strain peaked at the 5-h mark and afterwards consistently dropped, the wildtype kept increasing to a significantly higher OD_600_ before dropping at some point after the 8-h mark. At their highest, the wildtype reached an average OD_600_ of around 3.3. In comparison, the ∆*spo0A* strain topped at an average OD_600_ of 2.4.

For both strains, the cultures had all significantly dropped in OD_600_ after 24 h of growth, although it appears that the wildtype strain cultures generally still reach a higher OD_600_ compared to that of ∆*spo0A*.

### Microscopic characterization of *P. thermoglucosidasius* ∆*spo0A*

**Fig. 7:**
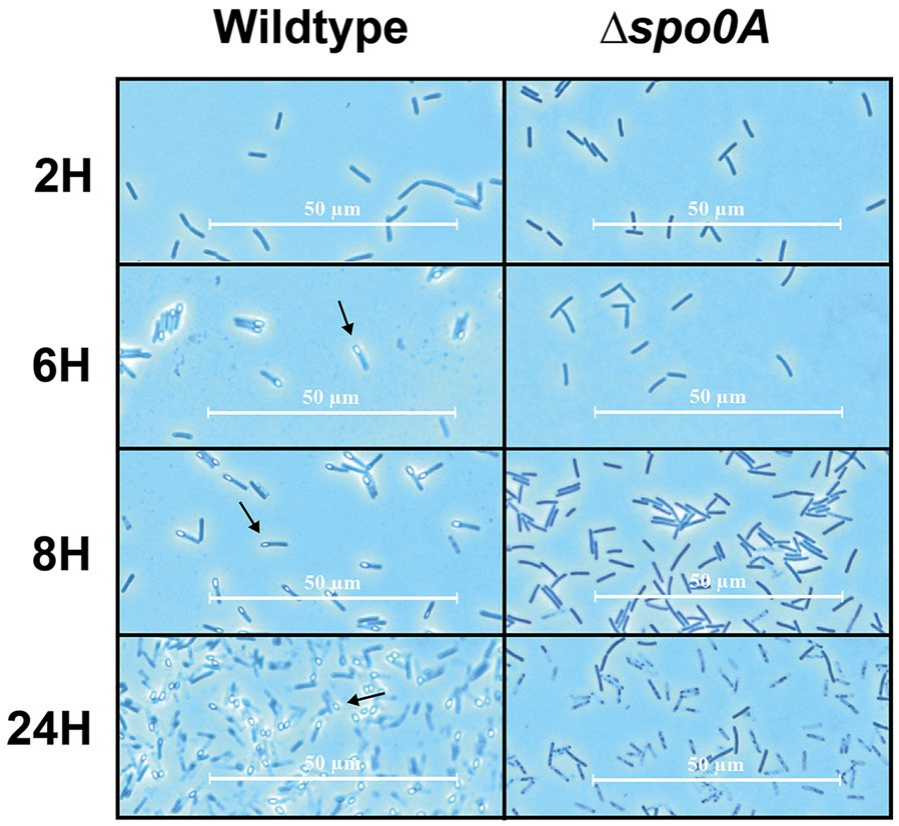
Phase contrast microscopy of samples collected from *P. thermoglucosidasius* ∆*spo0A* and wildtype cultures after 2, 6, 8, and 24 h of growth. During the exponential phase, at the 2 h sample, none of the strains appeared to be sporulating. However, after reaching stationary phase, at around 6 h of growth, the wildtype strain was producing large amounts of endospores, visible as bright bulbs located at the outer edge of the cells (examples indicated by arrows). No signs of sporulation was observed from the ∆*spo0A* culture

During the growth assay, samples were collected for microscopy at various growth stages of the cultures, in order to visualise the process of sporulation in both strains (Fig. 7).

The first set of samples was collected after 2 h, while the cultures were still in the exponential growth phase. Predictably, none of the cultures displayed any visual signs of sporulation, as they had not yet reached the point of nutrient starvation. The second set of samples was collected after 6 h—around the point where it appeared that growth had begun to stagnate. Here, it was observed that the wildtype strains expectedly had initiated sporulation, as there were clearly visible presences of endospores within the majority of the cells. In addition, many cells were also observed to have a slightly ‘top-heavy’ shape, indicating the beginning formation of a spore. Meanwhile, the ∆*spo0A* cultures did not display any visible signs of sporulation.

From the 6-h samples and onwards, the results of microscopy examinations remained consistent for all cultures. While the wildtype would continue to produce abundant amounts of endospores, none of the ∆*spo0A* cultures produced anything that visibly indicated the activation of sporulation. After 24 h, the wildtype and ∆*spo0A* cultures all showed significant signs of stress, appearing granulated and elongated. Nevertheless, this stress did not appear to have been able to push the ∆*spo0A* strain towards initiation of sporulation.

### Heat resistance assay

**Fig. 8:**
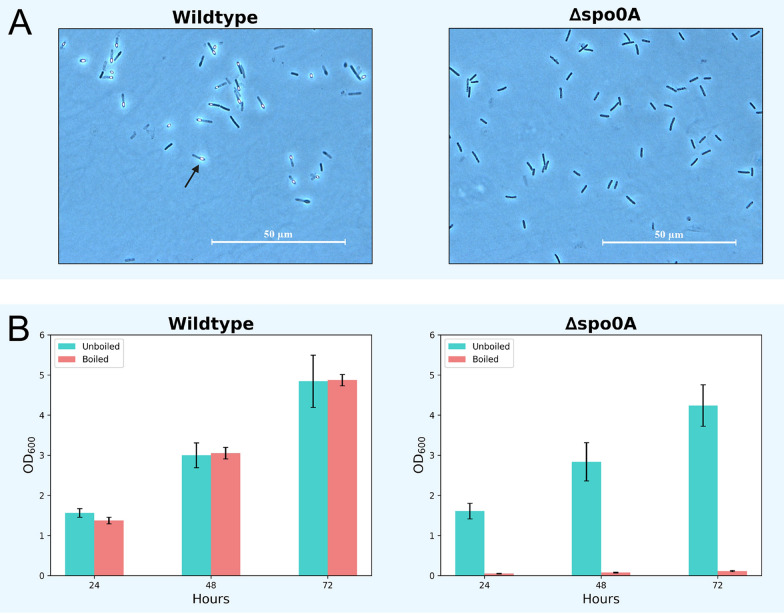
**A** Phase contrast microscopies displaying the state of the strains immediately before being boiled. The wildtype cultures were all visibly sporulating (arrow), while no signs of sporulation were detected in the ∆*spo0A* cultures. **B** OD_600_ measurements from cultures of wildtype and ∆*spo0A P. thermoglucosidasius*. Three replicates of each strain were allowed to grow to the point of nutrient starvation. The starved cultures were then boiled and transferred to rich medium for reculturing. After 24 h, the boiled wildtype cultures had grown to OD values comparable to those of the unboiled controls. In comparison, the boiled ∆*spo0A* cultures did not appear to recover within 72 h of culturing, even as the unboiled controls grew readily

One issue with investigating sporulation through microscopy, is that it can be significantly more difficult to visually detect lower concentrations of spores. We therefore decided to perform a secondary sporulation assay that screened for the presence of spores by taking advantage of their high heat resistance. As part of this assay, nutrient-deprived cultures of both *P. thermoglucosidasius* ∆*spo0A* and the wildtype strain were boiled in order to kill all vegetative cells, allowing only the spores to survive. The boiled cultures were then made to recover in rich medium, where any present spores would germinate and grow. Based on the growth, it could then be determined if any heat resistant spores had been present in the original culture. Prior to heat treatment of the cultures, it was confirmed through microscopy that the wildtype strain had induced sporulation, as predicted from earlier experiments. Consistent with previous observations, the ∆*spo0A* strain still did not appear to display any immediate signs of sporulation (Fig. 8A).

After recovery of the wildtype cultures, it was found that all inoculates of the boiled cultures were quick to reach cell density values comparable to those of the unboiled controls (Fig. 8B). This suggests that a high concentration of spores was present in the samples—something that was also confirmed through microscopy. In contrast, none of the ∆*spo0A* cultures were able to grow to significant ODs following heat treatment. As the unboiled ∆*spo0A* cultures grew without any apparent issues, it can be reasoned that the lack of growth of the boiled samples indicate that there were no spores present to survive the boiling.

In summary, the high degree of heat resistance in the wildtype cultures can be attributed to the presence of spores, which are capable of surviving exposure to high temperatures. Consequently, the lack of heat resistance in the ∆*spo0A* cultures suggests that there were few or no viable spores, presumably due to a lack of sporulation capacity.

## Discussion

To improve the genetic tools available for *P. thermoglucosidasius*, the present study demonstrates an improved shuttle vector that streamlines the selection process of *P. thermoglucosidasius* recombinants through the addition of a thermostable fluorescent marker. It is common for integration-based gene deletion strategies to identify double recombinants by screening for the loss of antibiotic resistance (Leer et al. 1993; Taylor et al. 2008; Cripps et al. 2009; Huang and Wilks 2017). However, this requires an extra culturing step, where the loss of resistance is determined by plating the colonies on medium with and without the antibiotic in question in order to identify sensitive colonies. In comparison, GFP-based vectors circumvent this extra culturing step, as the cells containing the plasmid generate a direct visual cue that makes them easily discernible from any double recombinants. Furthermore, since non-fluorescent colonies can be easily distinguished in a full plate of fluorescent colonies, less subculture-passes are needed to enrich the population of double recombinants, saving several days in the identification of the double recombinants. As such, the presented vector reduces both time and labour requirements for genetic manipulations of *P. thermoglucosidasius*. With the integration of an additional selectable marker, our optimised shuttle vector thus presents an improvement to traditional integrative shuttle vector-based modification that allows for generation of marker-less mutants. As the principles of this modification strategy has been long established in *P. thermoglucosidasius* (Cripps et al. 2009; Zhou et al. 2016; Sheng et al. 2017), our vector provides a simple and reliable genetic engineering approach, with a workload comparable to those of current alternative strategies. However, for more stringent selection it may be advantageous to rely on systems that utilise counter selection, such as those based on CRISPR/Cas9 (Lau et al. 2021).

Regarding the deletion of *spo0A*, the system produced several non-fluorescent colonies, from which around 70% of screened colonies appeared to be successful deletions. However, it should be taken into consideration that the efficiency of the deletion system will always be highly dependent on the target gene. If the target gene serves an important function, cells that successfully perform the second recombination will be more likely to die or be outcompeted by wildtype revertants. In these cases, it is to be expected that a higher number of non-fluorescent colonies will have to be screened in order to find the desired mutant.

### Impact of spo0A-deletion in *P*. *thermoglucosidasius*

It was decided to remove *spo0A* from *P. thermoglucosidasius* based on the assumption that this would affect the ability of the strain to sporulate. As such, tests were performed to determine what consequences the deletion had for the phenotype of the strain. In general, the *spo0A*-deletion appear to be highly effective at suppressing sporulation. Microscopy during the growth assay indicated that ∆*spo0A* cells do not produce spores, even when stressed during stationary phase. Supporting this, the heat resistance assay produced no indications that spores were present in the nutrient-deprived ∆*spo0A* cultures. Based on these results, it seems probable that the *spo0A*-deletion causes full repression of the sporulation pathway, rather than just limiting the expression. As such, *spo0A* is likely to be a favourable target for sporulation-suppression in *P. thermoglucosidasius* production strains, as even partial initiation of sporulation during fermentation would pose problems in large-scale production settings.

During the growth assay, it was observed that the growth rates of the ∆*spo0A* strain were largely similar to those of the wildtype strain. This is an advantage, as it would be preferable for a sporulation-free variant of *P. thermoglucosidasius* to maintain the high growth capacity of the wildtype strain. However, the growth assay also revealed that the wildtype strain reached significantly higher OD_600_-values before the point of stagnation. When working with spore-forming species, it is not unusual to attribute sudden OD drops around the stationary phase to the process of sporulation. However, as previously stated, sporulation appears to be fully suppressed in the ∆*spo0A* strain. As such, it is notable that the ∆*spo0A* strain still decreases to OD_600_-values that are equal to or lower than the wildtype strain.

There are several potential explanations for the discrepancy in maximum OD values between the two strains. In *B. subtilis*, the Spo0A protein is known to influence the expression of hundreds of genes, of which many are linked to the activation of sporulation (Molle et al. 2003). The removal of such a significant regulator is almost guaranteed to leave an impact on the regulatory network of the cell. It is not unreasonable to speculate that the deletion of *spo0A* could significantly shift the expression of a large number of genes in *P. thermoglucosidasius*, and that the early OD drop is a consequence of this change. A study by Tännler et al. (Tännler et al. 2008) found ∆*spo0A* to be unfavourable for sporulation suppression in riboflavin producing *B. subtilis*, as the deletion appeared to strongly increase the maintenance metabolism of the strain. Another study by Wang et al. (Wang et al. 2020) also observed that *B. subtilis* ∆*spo0A* displayed decreased biomass production during fermentation. However, they noted that multiple pathways involved in enzyme synthesis and secretion were upregulated, eventually concluding that the ∆*spo0A* phenotype could have a high capacity for enzyme production (Wang et al. 2020). In general, it is difficult to predict the full impact a deletion of a key regulator such as *spo0A* may have on cell metabolism, particularly in the case *P. thermoglucosidasius*, where much of the metabolic network has yet to be uncovered. Currently, it is unclear whether the differences in OD occur due to regulatory changes or something yet to be recognised. More research will have to be performed to fully determine the cause.

Based on the results of the present study, *spo0A* appears to be a viable target for sporulation removal in *P. thermoglucosidasius*, as it was not possible to demonstrate the induction of sporulation in the ∆*spo0A* strain. Still, it is important to acknowledge that the experiments performed in this study focused on induction through nutrient starvation, and thus did not consider any other stresses which could potentially trigger sporulation. As such, this study cannot preclude that changes to medium, culturing conditions, time scale, and other similar variables will not induce sporulation in the ∆*spo0A* strain. It is also possible that *P. thermoglucosidasius* could mutate in a way that circumvents the *spo0A* gene, and thus create a ∆*spo0A* strain which is still be able to activate the sporulation pathway. However, if the sporulation pathway of *P. thermoglucosidasius* truly resembles that of *B. subtilis*, then this should be considered unlikely, as a key transcriptional regulator presumably would be difficult to substitute. Nevertheless, there is still much to be learned about sporulation in *P. thermoglucosidasius*, and it will take more research in larger scale to truly confirm that the deletion of *spo0A* is the best way to ensure robust suppression of the sporulation pathway.

## Data Availability

The improved vector pMM7 (pGB-sfGFP-best carrying flanking regions of *spo0A*) has been deposited and is readily available at addgene to be used as template by any laboratory working on *P. thermoglucosidasius* genetic engineering. All data are available by request to the authors.

## Abbreviations

TMM: Thermophile Minimal Medium
SSS: Six Salts Solution
TSA: Trypticase Soy Agar
